# Key aspects of psychosocial needs in palliative care - a qualitative analysis within the setting of a palliative care unit in comparison with specialised palliative home care

**DOI:** 10.1186/s12904-023-01227-z

**Published:** 2023-07-21

**Authors:** Cathrin Michel, Hannah Seipp, Katrin Kuss, Michaela Hach, Andrea Kussin, Jorge Riera-Knorrenschild, Stefan Bösner

**Affiliations:** 1 Department of General Practice and Family Medicine, Philipps- University Marburg, Karl-von-Frisch-Straße 4, 35032 Marburg, Germany; 2 Professional Association of Specialised Palliative Homecare in Hesse, Wiesbaden, Germany; 3 Department of Anaesthesia and Intensive Care Therapy, Philipps-University of Marburg, Marburg, Germany; 4 Cilinic of Hematology, Oncology and Immunology, Philipps-University of Marburg, Marburg, Germany

**Keywords:** Palliative care, Palliative care unit, Specialised palliative home care, Quality of health care, Needs assessment, Qualitative research.

## Abstract

**Background:**

The number of palliative care patients with complex needs is increasing in developed countries. In addition to physical aspects and symptom control, psychosocial aspects are of great importance for palliative care patients. The aim of this study was to understand which psychosocial aspects are important to patients, relatives and health professionals within the setting of a palliative care unit in comparison with specialised palliative home-care (SPHC).

**Methods:**

We used a qualitative design based on semistructured interviews, which were coded via qualitative content analysis. The study took place in the state of Hesse, Germany, and data collection was conducted in 2017 (interviews from the ELSAH study, which was conducted in a SPHC) and 2018 (supplementary interviews conducted in a palliative care unit). The results from both settings were compared.

**Results:**

In the palliative care unit, 10 health professionals, 11 patients and 8 relatives were interviewed. In the outpatient setting, we interviewed 30 health professionals, 14 patients and 14 relatives. We identified four key psychosocial issues related to palliative care that were relevant in both the inpatient and outpatient settings: care planning, patient-centred care, a protected environment with feelings of safety, and psychological well-being. In addition, immediate availability of medical staff, greater relief of the relatives and better accessibility of psychological care were more important in the inpatient setting than in the specialised palliative home care setting.

**Conclusions:**

Knowledge and application of the identified key issues may improve patient-centred palliative care. Accessibility of psychological care and immediate availability of medical staff may be important factors for enhancing psychological well-being in the inpatient palliative care setting. Consideration of the identified key issues may help to develop more collaborative transitions between the palliative care unit and the SPHC and may help to provide palliative care patients and their families with care that is appropriate and feasible for them.

**Trial registration:**

The underlying comparative study of the outpatient setting of specialised palliative home-care (ELSAH) was registered within the German Clinical Trials Register DRKS-ID: DRKS00012421, (https://drks.de/search/de/trial/DRKS00012421) on 19th May 2017.

**Supplementary Information:**

The online version contains supplementary material available at 10.1186/s12904-023-01227-z.

## Background

Palliative care is a concept of holistic care for patients with life-limiting diseases that aims to enhance their quality of life and to provide relief from physical, psychological, social and spiritual suffering. Palliative care is complex, and it is important to provide comprehensive care for these patients, regardless of their illness or type of care [1–3].

Palliative care modalities in outpatient or inpatient settings and in specialised units vary across countries [4]. In Germany, palliative care can be provided at home as general or specialised outpatient palliative care (SPHC), in a hospital, in a nursing home, or in a hospice [5]. Regarding outpatient palliative care, there are two different structures: general palliative care and SPHC. Whereas general outpatient palliative care is provided by general practitioners and nursing services, SPHC teams have special additional training and work together in an interdisciplinary manner. Palliative patients with complex symptoms and care needs are eligible for specialised palliative care [6]. If additional hospitalization is needed, there is an indication for admission to the palliative care unit [7].

The number of palliative care patients with complex needs is increasing in developed countries as a result of population aging, the incidence of long-term cancers and the incidence of chronic diseases [8]. As a result, the needs for inpatient palliative care and outpatient care through SPHC teams are also growing [6].

In the ELSAH study [9], which is the underlying study to the comparison reported herein, aspects of successful SPHC and implications for quality assessment were obtained. Comprehensive care, treatment of complex symptoms, sense of security, facilitation of self-determination, a focus on the quality of relationships and respect for individuality were shown to be important factors for successful SPHC in the ELSAH study [9].

Previous studies indicate that in addition to physical aspects and symptom control, psychosocial aspects are of great importance for palliative care patients [10–12]. For example, psychological distress occurs in approximately 50% of palliative care patients [13, 14], and security, reliability, and the provision of appropriate resources are regarded as important for successful care [12, 15].

Respectful and compassionate care from experts, optimal communication, valued family involvement and care planning, ensuring patient safety and family involvement in care planning are regarded as key domains of importance for optimal hospital-based palliative care [16–18].

While several previous studies have focused on disease-specific patient care [19, 20], our analysis aims to understand which psychosocial aspects of care are important for palliative care patients, their relatives, and practitioners by considering all these groups independent of the underlying disease.

The aim of this study was to understand which psychosocial aspects of care are important to patients, relatives and health professionals in everyday hospital life and in the outpatient setting of SPHC and to examine how the psychosocial aspects differ between these two settings. Therefore, we did not define psychosocial symptoms beforehand, but extracted them from the analysis of the interview answers. Understanding these issues and differences might be beneficial in improving palliative care.

## Methods

### Study design and target group interview guides

This qualitative study [21] was conducted at the palliative care unit of the Philipps University of Marburg, Germany, and in the SPHC setting in the state of Hesse, Germany. This study evolved from the findings of the ELSAH study [9]. The study was conducted in accordance with the Consolidated Criteria for Reporting Qualitative Research (COREQ) checklist [22].

We aimed to determine whether patients, relatives and professionals in an inpatient setting of a palliative care unit experience psychosocial aspects of care in a similar or different way than individuals in outpatient settings. For this purpose, interviews in the inpatient setting of a palliative care unit were conducted and compared with interviews from the ELSAH study [9].

Based on the interview guides published by Seipp et al. [9], three semistructured interview guides were developed for interviews with health professionals, patients, and relatives (Supplemental Material 1) in the palliative care unit.

### Recruitment and data collection

Data were collected from July to October 2018 in the palliative care unit and from May to November 2017 in the SPHC setting. Semistructured interviews were performed with health professionals, patients and relatives in both settings. In both settings, we purposively sampled [23] to achieve a balance between health professionals, patients and relatives.

All interviewees had to be at least 18 years old, to be able to speak fluent German and to be able to give informed consent. Patients were classified according to their family status. To be eligible, patients had to be receiving either specialised palliative home care in the SPHC setting [9] or inpatient palliative care in the palliative care unit. All different palliative diagnoses have been accepted.

Health professionals either worked in the palliative care unit (inpatient setting) or worked in or with SPHC (outpatient setting).

Relatives were identified by the patients themselves or by professionals in the palliative care unit or the SPHC setting.

Semistructured interviews were conducted face-to-face in a separate room of the palliative care unit or the patient‘s room. If the patient and his or her relative participated, the patient could decide whether the interviews would be conducted together or separately.

For the comparative data collected in the SPHC study [9], semistructured interviews were conducted with patients, their relatives and health professionals.

We audio-recorded the interviews and made additional field notes.

HS (M.Sc. Public health, female, 6 years of experience) and KK (M.Sc. Physiotherapy and Health Scientist, female, 21 years of experience) conducted the interviews in the SPHC setting; CM (physician, female, 4 years of experience) conducted the interviews in the setting of the palliative care unit. The interviewers and participants did not have any relationship before the study. Participants were informed about the goals of the study.

After completing the interviews, participants completed a demographic form.

Recruitment of patients and relatives was carried out upon health professionals’ proposals. Participants were approached face-to-face in the palliative care unit. In the SPHC setting, professionals were contacted via telephone, whereas patients and relatives were approached by SPHC employees, who passed on the contact details to the research team. No other individuals were present during data collection. The transcripts were not returned to participants.

The end point of data collection (sample size) was not determined a priori but was set upon reaching the saturation point with a lack of new arguments and new aspects.

### Data analysis

After audio-recording, verbatim transcription of interviews was performed using the software f4transcript, version 7 [dr.dresing&pehl GmbH, Marburg, Germany] [24]. All interviews were pseudonymized [24, 25].

We coded the interviews using MAXQDA, versions 2018/2020 [VERBISoftwareConsult, Berlin, Germany] [26]. The coding tree was derived deductively from the interview guide and supplemented inductively by newly emerging aspects.

Our coding tree (see Supplementary Material) was based on the analysis we had performed in the outpatient SPHC setting [9]. As new topics were added in the inpatient setting, the coding tree was modified and discussed among CM, HS and SB (M.D., senior health scientist, male, 15 years of experience) until consensus was reached. Subsequently, we analysed the data according to the new coding tree.

Coding was performed independently by the authors, and then, they compared their results. CM performed coding in both settings. We did not aim to be uninfluenced during the coding in the inpatient setting; instead, we looked for similarities and differences between the two settings during the coding, as that was research goal.

Codes and subcodes were analysed as described by Kuckartz et al. [27]:

We read the text passages of the categories with the help of MAXQDA software. Thus, we were able to depict rating clusters and to describe significant findings. Summarized text passages of the codes were described for the evaluation and placed in the overall context.

By discussion within the team, we reviewed our findings until consensus was reached. Participants did not provide feedback on the findings.

## Results

In the palliative care unit, 10 health professionals, 11 patients and 8 relatives were interviewed. The interviews lasted from 19 to 58 min. One interview was terminated prematurely after 5 min, as the interviewee was not able to continue due to health reasons.

In the outpatient setting, we interviewed 30 health professionals, 14 patients and 14 relatives. The interviews lasted from 23 to 98 min. Due to health reasons, two patients and two relatives withdrew their consent to be interviewed.

No individual was interviewed more than once.

The characteristics of all participants are classified in Table 1. More detailed characteristics of patients, relatives and health professionals are shown in Tables 2 and 3.

**Table 1:** Participant characteristics

Setting		Palliative care unit			SPHC		
Participants		Patients	Relatives	HP*	Patients	Relatives	HP*
Number, n		11	8	10	14	14	30
Gender; n (%)	Female	5 (45.5)	6 (75)	7 (70)	8 (57.1)	9 (64.3)	22 (73.3)
	Male	6 (54.5)	2 (25)	3 (30)	6 (42.9)	5 (35.7)	8 (26.7)
Age; years; median (range)		70 (54–85)	59 (45–71)	46 (33–61)	64 (38–83)	63 (23–78)	54 (26–67)
Nationality; n (%)	German	11 (100)	6 (75)	9 (90)	13 (92.9)	13 (92.9)	30 (100)
	Belgian	0	0	1 (10)	0	0	0
	Romanian	0	1 (12.5)	0	0	0	0
	Turkish	0	1 (12.5)	0	1 (7.1)	1 (7.1)	0

*Health professionals

**Table 2:** Detailed information regarding patients and relatives

Setting		Palliative care unit		SPHC	
Participants		Patients	Relatives	Patients	Relatives
Number; n		11	8	14	14
Main diagnosis; n (%)	Cancer	10 (90.9)	n/a	12 (85.7)	n/a
	Cardiovascular disease	1 (9.1)	n/a	2 (14.3)	n/a
Family status; n (%)	Married/living with partner	8 (72.7)	6 (75)	9 (64.3)	12 (85.7)
	Single	1 (9.1)	2 (25)	2 (14.3)	1 (7.4)
	Widowed	2 (18.2)	0	2 (14.3)	1 (7.4)
	Divorced			1 (7.3)	0
Relationship to patient; n (%)	Spouse or life partner	n/a	5 (62.5)	n/a	7 (50)
	Child	n/a	1 (12.5)	n/a	2 (14.3)
	Sibling	n/a	1 (12.5)	n/a	2 (14.3)
	Cousin	n/a	1 (12.5)	n/a	0
	Parent			n/a	1 (7.1)
	Grandchild			n/a	1 (7.1)
	Bereaved Spouse			n/a	1 (7.1)
Frequency of contact	Every day	n/a	7 (87.5)	n/a	13 (92.9)
to patient	4–6 days/week	n/a	1 (12.5)	n/a	1 (7.1)

n/a – not applicable

**Table 3:** Professions of interviewed professionals

Setting		Palliative care unit	SPHC
**Number; n**		10	30
Profession; n (%)	Nurse	3 (30)	15 (50)
	Physician	3 (30)	7 (23.3)
	Psychologist	1 (10)	0
	Social worker	1 (10)	1 (3.3)
	Pastor	2 (20)	3 (10)
	Home-care hospice service coordinator	0	4 (13.3)

Eleven codes and 64 subcodes were generated from the interviews with the health professionals. Ten codes and 45 subcodes were generated from the interviews with the patients and the relatives (see Supplemental Material 2) in this analysis.

Herein, we present the psychosocial aspects that were deemed to be important by the participants in the inpatient and outpatient settings. The results are supported by example quotes from the interviews.

### Care planning

It was important for all patients in the inpatient and outpatient settings to gain clarity regarding their care perspectives and to receive support in this matter.

In the palliative care unit, the planning of follow-up care is carried out with the help of the social service, which shows the patients’ and their families’ different future perspectives. This includes advice and the organization of proposed measures. All patients and relatives considered very useful that the social service of the palliative care unit organized aides that are needed when patients plan to move to the home setting.

*“There is a woman here, I have unfortunately forgotten her name. She organizes everything in the background. She contacts the palliative care team on site. And they coordinate and consult with each other, and she discusses the details with the patient. That has already happened. What equipment and aides are still at home, what is already available, and what is still missing.” (3205 A, relative, female, palliative care unit)*.

*Patient: “Up to now I have still managed alone. But now I don’t think I’ll be able to do everything alone.”*

Interviewer: “And have you already spoken to someone here on the ward?“

Patient: “Yes, that is to be organised.“

Interviewer: “Do you know what will be organised?“

*Patient: “The nursing service is supposed to come, and I also have someone from the palliative care team.” (3101P, patient, female palliative care unit)*.

All employees of the palliative care unit regarded care planning as an important task.

“*Exactly, and when it comes to discharge, then just all the practical things. Possibly apply for a care level or a higher classification. Organizing aides. Ensuring that the SPHC gets on board. I’ve already told you about aides. That everything they need is at home. That a nursing service comes. If they need oxygen, that’s at home. If they need parenteral nutrition, there is a company that takes care of it. That it is always brought. Exactly, and then to document everything.” (3510E, social worker, female, palliative care unit)*.

Organizational relief was also a major concern for the staff of the SPHC teams. Establishing networks with service providers enables SPHC teams to provide help quickly and uncomplicatedly. In contrast to inpatient care, the planning of care by the SPHC team takes place in the home setting and the familiar everyday environment, which patients and relatives find relieving. The presence of an expert who looks at the situation from an outside perspective and then organizes the necessary support was felt to be helpful.

*“Yes, as I said, the application for the level of care needed and: ‘Why don’t you have a commode chair? You can use it for all kinds of things!’ And: ’Do you still need it? Do you need these medications?’… She (SPHC nurse) knows a lot about medication and everything. …When she was with us and it was a matter of some medication and so on, she called…and then it was quickly clarified. … As an outsider, of course, you see this operating blind. We both at home, all day at home in our rut, of course do not see many things that an outsider then sees! And that was very good when she came!” (1308 A, relative, male, SPHC)*.

*“Let’s say outpatient care services, all these organisational questions are also taken care of by this palliative team, for the most part, I don’t want to say taken care of, but at least initiated. And there I have people who know me, my situation, my living situation, who can also assess this. With me, “What is the right thing to do now?“ (1316P, patient, female SPHC).*

In particular, the organization of medications and aides as well as support in filling out applications was perceived very helpful by patients and relatives.

*“And organization is actually the most important thing after symptom control. That everything is there. And that everything runs well somehow.” (1517E, nurse, female, SPHC)*.

It was important to the staff of the palliative care unit and SPHC to plan care with the best interest of the patient in mind, to respect his or her will as much as possible, and to include the wishes of the relatives.

*“So, when patients express wishes, even if they are not adequate or feasible, we take the time to sit down with the patients or their relatives and discuss them sensibly. That is often not possible in other wards. Yes. That you really take the time.” (3502E, physician, female, palliative care unit)*.

### Patient-centred care

For all staff of the palliative care unit and SPHC, it was important to focus on the patient and his or her wishes. This was attempted to be implemented in everyday care through the following aspects: welcoming culture, having time, relief, preparation for the end of life, empowerment and communication.

It was important to the staff on the palliative care ward to create a welcoming culture for relatives and to give them the freedom to decide whether they would like to stay on the ward and spend the night or not (after consultation with the patient). There were no time limits for visits to the ward, but rather a framework of security was created in which the relatives could feel invited and included. This was gladly accepted by the relatives, and all relatives expressed positive feelings about the ability to be with their relatives.

*“That they just say, ‘Gee, I can come and go here whenever I want… I’ll take my coffee… I belong to this ward…I belong with my relative…. I’m allowed to take any drink… I’m allowed to take any snack… No one asks me!’ And when I say, ‘We’re going to go outside!’ Then we’ll go outside.” (3503E, physician, female, palliative care unit)*.

The palliative care unit was described by patients as a place that gave them the time to recover and to come to rest, to say goodbye to their relatives without the stresses of everyday life.

*“Here in the palliative care unit, I have peace and quiet. This allows me to relax and concentrate fully on what is necessary.” (3301P, patient, male, palliative care unit)*.

The treatment in the palliative care unit gives the relatives time for themselves to recharge and organize things that have been left undone, especially after a preceding exhausting period of care.

In SPHC centres, patients appreciate being able to continue to experience their daily lives with their family in a familiar environment rather than being treated in a hospital among strangers. For example, the preferred way of life, including food and meals at home in their familiar environments, made patients feel better. In this case, food intake reflects the well-being of patients.

*“All the hospital food, I couldn’t eat that! The devil knows! I came home and could eat, ne?” (1314P, patient, male, SPHC)*.

The relatives in SPHC state that they are happy to have their relatives with them and to be able to take care of them.

*“That you make it as nice as possible for him. That he (exhales loudly) Pfff! Yes. So, for him that was that he is at home.” (1901 H, relative, female, SPHC)*.

On the other hand, some of the relatives cite a resulting burden and organizational challenge in the SPHC setting.

*“We just try to organize and arrange the daily routine, or in my case with my professional activity, so that we can ensure care and attendance.” (1319 A, relative, male, SPHC)*.

All the staff interviewed in both the outpatient and inpatient settings deemed it important to have time for their patients and relatives, to recognize the hardships of these people, and to perceive their needs. This was also reflected by the patients not by placing their illness in the foreground but in taking time to understand their complaints and needs. The behaviour of the staff towards palliative care patients seems to make the palliative situation more bearable for patients and their families.

*“She (nurse) has time for me. She understands my sorrow and I think that’s very important.” (3205 A, relative, female, palliative care unit)*.

Psychosocial and organizational relief played a major role in caring for relatives on the palliative care unit; this also occurred through the relatives relinquishing responsibility for care, among other things. Psychosocial relief was provided through talks, support from the staff, psychological support, and interaction with other relatives of palliative care patients.

“*What is important for the relatives? Well, first of all, relief. That they can relinquish responsibility. Knowing that their relative is being well cared for. ‘I don’t want him to suffer.’ He or she. That is often, is often said. Also once again this time TO HAVE, this time somehow to say goodbye.” (3510E, social worker, female, palliative care unit)*.

In the outpatient setting, organizational relief was usually cited first as a major help. However, psychosocial support, especially through discussions in everyday care, was just as important, and both forms of relief made it easier for relatives to care for their family members at home. The SPHC staff emphasized that it was important to stabilize the relatives and their environment since they took over the main care and enable the patient to remain at home.

*“And how can I support the family? Because that is the supporting force. The family is the force that bears the greatest burden because the family is there in 24 hours. We come, do our work, stay with the patient for a while and leave again. But the family then gets the job of continuing to care for the patient. And for me it is very important not only to stabilize the patient so that he is well controlled, so that he still has some quality of life, but also to help the family so that they can bear this burden.” (1508E, nurse, female, SPHC)*.

### Protected environment and feelings of safety

The palliative care unit and the SPHC team both create a protected environment for patients. This enables patients to find peace and clarify perspectives for themselves.

An important aspect for patients and their relatives was safety: having a contact person at all times and promptly receiving the help they need.

*“Yes, that they all take care of me and do so. And also when you ring the bell at night and then you need something to drink or something and the nurses, they do it all so sweetly and nicely.” (3303P, patient, female, palliative care unit)*.

*“And if something’s wrong, I have the number and can call them. They would come at any time. That’s reassuring for me. To know if something’s wrong, he doesn’t want an ambulance and nothing. It could be something at night. I used to lie awake all night and didn’t know: “If something’s wrong, what are you going to do?“ And now I know: “Okay, if something’s wrong, you call the palliative care!“ And that is a relief for me. I can sleep again! I haven’t been able to for months. For years, in fact, almost not at all.” (1306 A, relative, female, SPHC)*.

The way in which a sense of security is conveyed to patients and family members differs between inpatient and outpatient palliative care settings.

For relatives on the ward, it was important that the patient was well cared for and did not suffer physically or emotionally and that a contact person was available at all times. This safety aspect in the inpatient setting led some palliative care patients to decide that they would rather die on the ward than at home.

*“It’s also, as patients always say, ‘Well, here I just have to push a button and someone will come.’ So, a maximum sense of security. That’s not even possible at home. At the push of a button.” (3501E, social worker, female, palliative care unit)*.

*“I find them in good hands here… And the staff are also very nice… That’s reassuring for them, too.” (3203 A, relative, male, palliative care unit)*.

Compared to the inpatient setting, where the caring expert is immediately available, in the outpatient setting, the patient must first cope with the care provided by the family member or, in an emergency, wait for the arrival of the SPHC team. However, the knowledge that the SPHC team can be reached by telephone at any time gives patients and relatives a high degree of security.

*“And if something happens, I have the number and can call them. They would come at any time. That’s reassuring for me“. (1305P, patient, male, SPHC)*

*“We have on-call services, and the patients can all dial this number at any time, 24 hours, at night or on Sundays then and say, “I have a problem!“ Or, “I’m not feeling well! And please come!“ Yes. This security is very important for them. For the relatives and also for the patients.” (1503E, nurse, female, SPHC)*.

### Psychological well-being

The psychological well-being of palliative care patients played a major role in both settings. It was essential for staff to have time for and a relationship with patients and relatives to be able to talk about psychological problems.

*“That you build up a basis of trust in a completely approachable, open, friendly way. And then a lot is possible. Yes!” (3505E, nurse, female, palliative care unit)*.

Anxiety was mentioned as a main issue, especially with pain and shortness of breath.

*“And in addition, there is a situation that she suffers very strongly from fears. Simply of the situation, of what can still come. Fears of pain that could occur. Especially in connection with the air. With the shortness of breath, that’s one of her main concerns.” (1319 A, relative, male, SPHC)*.

Among palliative care patients receiving SPHC and before discharge from the palliative care unit to the home setting, fear of being a burden to family members and of being alone was frequently mentioned. The fears differ across the two care settings.

*“That in the time maybe someone would come, who then stays with him. When he is alone, he is of course more afraid than when someone is with him.” (1304 A, relative, female, SPHC)*.

Common symptoms of palliative care patients mentioned both in the palliative care unit and in the home setting include sleep disturbance, tension, agitation, confusion, feelings of uselessness, depressed mood, and suicidal ideation.

*“As soon as I went to bed, it was over. As soon as I lay flat. It was bad! Very, very bad! I wanted to die! I really wanted to end, but then I was too cowardly….” (1102P, patient, male, SPHC)*.

The staff of the palliative care unit and the SPHC teams regarded the emotional support of patients, contact with relatives, a familiar environment and long-preserved independence as important factors in increasing the patients’ psychological well-being. Here, it was shown that the strongest influence on psychological well-being was symptom reduction, especially pain reduction.

*“I would be happy if I were free of pain and if I could live normally again.” (3101P, patient, female, palliative care unit)*.

A range of therapy options was available in the palliative care unit, depending on the severity of the symptoms. Patients and relatives appreciated the good availability and uncomplicated contact here. Low-threshold and short-term offers of conversation by nursing staff in everyday nursing care were seen as helpful for the patients’ own person and as an expression of appreciation. Some of the patients gladly accepted the offer of music and art therapy, relaxed in the process and the illness receded into the background.

*“I enjoyed that this morning with the music. Have I ten minutes, there I have, they can go another ten minutes. That was a real relaxation for the body. That was very good.” (3303P, patient, female, palliative care unit)*.

Patients and relatives usually found the talks with psychologists or pastors relieving, and the possibility of concluding topics, reflecting on life once again, and receiving help in coping with the illness from a person outside the family was seen as positive.

*“And the relatives also need a space from time to time where the patients are not present. So, if the patient is not present, they talk to us again. Then, sometimes we sit here. Then, sometimes we take a step outside with family members. And they take another breath or have a coffee or something. And they can also relieve themselves once again. In conversation or simply in being together with us.” (3506E, pastor, female, palliative care unit)*.

*“A psychologist was there a few days ago and offered to talk to Mrs. (X) [patient]. But Mrs. (X) did not want it. And she also mentioned that it was also for relatives and after this bad night. I have already thought about it, I have to think about it for myself. I talk a lot with my mum at home. It’s important to talk to someone. About the things that you don’t say here in front of Mrs. (X). But I have already considered this for myself. Yes, to talk to someone again. Who might be able to give me a few more tips.” (3205 A, relative, female, palliative care unit)*.

The staff of the SPHC team offered to talk to their patients and their relatives, asked questions, listened and tried to support them emotionally in this way. For some employees, conversations about psychological problems with patients were challenging, and they reached their personal limits. The team meetings and supervisions were helpful in these cases; this framework was also often used to decide whether further therapeutic help was needed for patients. Most staff members saw their role as noticing symptoms and problems, supporting patients within their own means, and addressing and mediating in the more difficult cases.

In the palliative care unit, more trained staff are available for patients and their relatives to talk about psychological stress. In the outpatient setting, it is often the relatives who are the first to address the psychological problems of palliative care patients. The employees of the SPHC teams significantly relieve the burden of those they care for by offering to talk to them. If there is an additional need for psychological care, well-networked structures are required in the outpatient area, as SPHC teams usually do not include their own psychologists.

*“I believe that we are able to provide a relatively high level of psychosocial support for patients and their families. Because, on the one hand, we have an open ear. In other words, we take care of their worries and needs. Even if it’s just a banal solution, but we try to offer a solution that people will cling to. And that then also has a psychosocially relieving effect. But if the cases are totally complex, where I don’t know what is connected with massive anxiety disorders, panic, then of course you have to try to fall back on the professional level. In the form of psycho-oncologists. Or psychiatrists, whatever. So, I notice that this is always a bit more difficult here than in a hospital, because there you simply called the psycho-oncology department and they were there. That’s just not so easy here.” (1516E, nurse, female, SPHC)*.

However, voluntary home hospice groups, which partly cooperate with the SPHC, can provide some psychological support in the SPHC setting, which is no substitute for professional psychological care.

*“We can be approached by anyone. So, often by relatives, sometimes by doctors, sometimes by hospitals. … We are often in old people’s homes, because that is where the people live, that if there is a need for psychosocial support for relatives or patients, then we are asked. We then go there… First of all, we look at what they need. … And then we look to see which of our volunteers, who are all trained in accompaniment, can do it, and who fits in. Because only when the chemistry is right can we say: “Yes, he or she can do that! … That they do something together, I don’t know, that they have conversations, that they…some go to town again and eat ice cream or go to the cinema or whatever. Sometimes the patient is not the one who needs support, but the relatives. That the wife has to go shopping, to the hairdresser or simply needs a break. That one stays there and fills that time. Sometimes there is also a need for conversation; that someone is there who wants NOTHING from you, but simply has time, is there and listens.” (1504E, member of a voluntary hospice group, female, SPHC)*.

## Discussion

### Main findings

We identified four psychosocial key issues that were important for patients, relatives and health professionals in a palliative care unit and in the setting of SPHC with slightly different foci, which were, among others, attributed to different contextual factors. Participants revealed that care planning, patient-centred care, a protected environment with feelings of safety and psychological well-being were central issues for successful palliative care.

These aspects are obviously interrelated, as the quality of one of these issues influences the others.

Our findings confirm results from previous studies in the inpatient palliative care setting, which have identified similar psychosocial key domains of high importance for patients and their families, including expert care, respectful and compassionate care, optimal communication, valued family involvement in care planning, environmental privacy for families and ensuring patient safety [16–18].

The key aspects identified in inpatient care resemble central issues collected in the setting of specialised palliative home care [9, 15, 28]. Our study showed that personal relationships are important both in the outpatient and inpatient settings of specialised palliative care and supported results from previous analyses in this regard [9, 12, 18].

However, in our inpatient palliative care unit, slight differences and accentuations became apparent when compared to the outpatient setting.

First, the immediate availability of medical staff and the associated immediate possibilities for intervention led to feelings of safety among patients in the palliative care unit. In the palliative care unit setting, relatives have the assurance that their family members will be well cared for immediately. In the SPHC setting, the patient primarily has to cope with care via relatives or, in an emergency, wait for the SPHC team to arrive. However, the 24-hour accessibility of the SPHC teams gives patients and their relatives a high degree of security. Such a sense of security has already been described as relevant for palliative care in previous studies [9, 29, 30], and the sense of security is also conveyed by nonmedical factors such as financial aspects and feelings of self-efficacy. Direct comparisons between in- and outpatient palliative care regarding sense of security have not been made thus far.

Second, both patients and their relatives expressed conflicting perceptions of the inpatient setting as a source of stress and relief compared with the home environment. On the one hand, patients and relatives often experience the palliative care unit as a place for relief and deceleration; on the other hand, there is a desire to be at home and to enable care at home. Previous studies have shown that patients in the early phase of a palliative illness prefer to die at home [31, 32]. Age, gender and socioecological factors seem to influence patients’ preferences for the place of death [33, 34]. Thus, female gender and living alone were negatively associated with home death [35, 36].

It might be concluded that the optimal places of end of life and death vary between individuals and that—among others—both a palliative care unit and the home environment can provide an adequate setting for the individual patient.

Third, regarding accessibility of psychology care, psychologists were directly available in the palliative care unit, whereas only a minority of SPHC teams had a psychologist on staff, and psychological problems were often first discussed with relatives in the outpatient setting. Previous studies of cancer patients have found that they deem the prompt availability and accessibility of psycho-oncological care to be important [37]. A study analysing psychological care in hospices in the United Kingdom showed that basic emotional support was considered sufficient, whereas overall service and more specialist psychological care were assessed as inadequate [38].

Further studies comparing accessibility and availability of specialist psychological care in palliative care units and SPHC are needed.

It might be useful for outpatient SPHC teams to build a network with psychologists or to increase the inclusion of psychologists in specialised palliative home-care teams.

### Strengths and limitations

The fact that participants from only one palliative care unit were recruited for reasons of practicability might have led to overestimation of site-specific issues. However, we have no evidence that this ward differs in any way from comparable wards in Germany.

The purpose of the paper was to gain an understanding of psychosocial needs in inpatient and outpatient palliative care settings from the insights, statements, and attitudes of the participants during the period of care. However, the different costs of care in the two settings were not examined or considered.

Neoplasms were the most frequent diagnoses of the patients included, which corresponds to the fact that the majority of palliative care patients in Germany are cancer patients [39]. The mean age of our patient collective was comparable to the mean age of palliative care patients in Europe [40].

However, with only three nononcologic patients in this study, a sufficient in-depth exploration in nononcologic patients was not possible.

One can argue that the analysis of the previously conducted SPHC interviews was not primarily designed for comparison with a palliative care unit.

After the interviews in the SPHC setting, however, it was evident that comparisons were often made with the inpatient setting, so we decided to proceed in this way.

Therefore, in the coding process, the codes from the ELSAH study that were not relevant to the question of psychosocial needs were removed, and other codes that emerged as new relevant topics during the interviews on the palliative care unit were added and codes were adapted, as the ELSAH study did not focus especially on psychosocial needs but included these, among others.

### Implications

Our study contributes to the finding that psychosocial aspects, care planning and a sense of security are important for successful inpatient and outpatient palliative care [1, 15, 41].

Similarities to other trials [16–18] suggest that our results are generalizable to other palliative care units.

In particular, it might be beneficial to expand psychological care in specialised palliative home-care teams.

## Conclusion

Four psychosocial key issues were described by patients, relatives and health professionals to contribute to the success of specialised palliative care: care planning, patient-centred care, a protected environment with feelings of safety and psychological well-being. These issues were of relevance in both the inpatient and outpatient settings. Immediate availability of medical staff, a possible greater sense of relief among relatives and wider accessibility of psychological care became evident in the inpatient setting when compared to specialised palliative home care. The differences between the individual settings enable the patients and their relatives to choose the appropriate setting and to change it if necessary (e.g., if the relatives are overburdened in the home setting or - vice versa - if the palliative care patient wishes to spend the last days at home with the support of the SPHC team) and to receive the support that is suitable for them at that time. It is important to have different settings to be able to offer individual, patient-centred care.

## Electronic supplementary material

Below is the link to the electronic supplementary material.

Supplementary Material 1

Supplementary Material 2

## Data Availability

For data protection reasons, no original data can be made available. Supplemental material on data collection and analysis is provided.

## List of abbreviations

ELSAH: Evaluation of specialised palliative home care by the example of Hesse
HP: Health professional
n/a: Not applicable
SPHC: Specialised palliative home care
