# Supplementary material for: Key aspects of psychosocial needs in palliative care - a qualitative analysis within the setting of a palliative care unit in comparison with specialised palliative home care
Source: BMC Palliat Care. 2023 Jul 21;22:100. doi: 10.1186/s12904-023-01227-z (PMC10360287; doi:10.1186/s12904-023-01227-z)
Supplement: Supplementary file 1 — Supplementary Material 1 [file 12904_2023_1227_MOESM1_ESM.docx]

**Supplemental material**

1. **Semi-structured interview guides (translated into English)**

**Note:** Interview guides served as checklists. The interviews were semi-structured and guided according to the narrative flow of the interview.

- 1. **Interviews with health professionals**
- **Please tell me something about your professional background!**
- What made you want to work in the palliative care unit/ in palliative care?
- **For what reasons are patients admitted to the palliative care unit?**
- **In your view, what constitutes successful palliative care? (Ideal)**
- **What needs do patients and family members have besides symptom control**? (Psychological, social, spiritual, ethical)
- How are the needs identified?
- What do you think is the most important thing for patients and relatives in the palliative care unit?
- Can you please describe how patients and family members can influence care?
- **Comparing the care provided in the palliative care unit to specialised palliative home-care, do you see differences in terms of important aspects of care?**
  - What is special about the palliative care ward? (In comparison to specialised palliative home-care or to the normal ward?)
  - Can you describe how care is adjusted as it progresses?
- **Can you describe instances where something didn't go well in care? (In comparison to a good course)**
- **What goals would you like to achieve with your work?**
- **As a team, you discuss each case. How do you structure that? What all should be discussed?**
  - How do you go about formulating goals or clarifying the mission of those affected?
- **How is dying addressed with patients and family members? (Wishes regarding location, environment)**
- **As a palliative care patient, where would you like to die and why?**
- **Finally, do you have any suggestions/criticisms/other issues you would like to address?**

- 1. **Interviews with patients**
- **Would you like to tell me how you are doing at the moment?**
- **What is most important to you right now?**
- **What helps you the most at the moment? (medical/non-medical).**
  - What could support you additionally?
  - To what extent can you influence supply?
  - What could be better from your point of view?
- **Can you describe the support you receive in the palliative care unit? (Areas of life)**
- **Who decided that you should be stationary?**
  - Why were you admitted as an inpatient?
  - What was it like for you when you heard you were going to be admitted to the palliative care unit?
  - What has changed since you were admitted as an inpatient?
- **What is your living situation?**
  - Who is involved in home care?
- **How are you physically?**
- What does physical and emotional well-being mean to you at the moment?
- How satisfied are you with your physical and emotional well-being at the moment?
- **How is your home environment involved in care?**
- **What is most important to you right now for your loved ones?**
- **What gives you pleasure?**
  - In your current situation, what helps make your life as livable as possible?
- **Would you like to talk to someone about your situation?**
- **How do you deal with your situation? (Coping strategies?)**
- **What gives you strength?**
- **Would you consider yourself a believer in the broadest sense? (religious/spiritual)**
  - Are the beliefs you spoke of important to your life and to your current situation? Does your faith sustain you?
  - What role should this play in care?
- **What thoughts do you have about dying? (Place, environment)**
- **Finally, do you have any suggestions/criticisms/other issues you would like to address?**

- 1. **Interviews with relatives**
- **Would you like to tell me how you are doing as a loved one right now?**
  - Do you want to tell me how your loved one is doing right now?
- **What's most important to you right now?**
- **What is most important to you for your loved one right now?**
- **What helps make your loved one's life as livable as possible?**
- **What complaints (symptoms) are currently causing your loved one the most problems?**
- **How quickly do his/her complaints change?**
- **Who all is currently involved in the care? Caring for your loved one? (Professional groups, other people)**
- **From your perspective, who determines what medical or other interventions the patient gets?**
  - What additional support would you want for your loved one? (Medical/ non-medical)
  - To what extent can you have a say in what medical or other procedures the patient receives?
- **What does it mean to you that your loved one has been admitted as an inpatient?**
- **How do you rate the conditions in the palliative care unit? (Visiting hours, responding to individual wishes)**
- **In which places could something go better?**
- **Finally, do you have any suggestions/criticisms/other issues you would like to address?**

1. **Coding trees (original in German)**
   1. **Interviews with health professionals**

| **Code** | **Number of Codings** |
| --- | --- |
| 1. Experte | 0 |
| - 1. Aufnahme | 18 |
| - 1. Person und Beruf | 63 |
| - 1. Motivation, Einstellung | 116 |
| - 1. Eigene Be-/Entlastung | 108 |
| 1. Palliativstation und SAPV | 0 |
| - 1. Team Charakteristika | 169 |
| - - 1. Teamarbeit, Teamstabilität | 62 |
| - - 1. Teaminterne Evaluationen | 49 |
| - - - 1. Selbstreflexion | 15 |
| - - - 1. Retrospektiv | 23 |
| - 1. Zusammenarbeit | 5 |
| - 1. Probleme | 41 |
| - 1. Vergleich | 24 |
| - 1. Interdisziplinarität | 43 |
| 1. Besonderheiten Palliativstation | 41 |
| 1. Besonderheiten SAPV-Arbeit | 2 |
| - 1. Erstassessment | 43 |
| - 1. Indikation SAPV | 39 |
| - 1. Verlauf | 49 |
| - 1. Anlass und Frequenz | 25 |
| - 1. Netzwerk, Koordination | 65 |
| - - 1. Regelversorgung | 72 |
| - - 1. Hospiz / amb. Hospizdienst | 70 |
| 1. "Tätigkeiten" | 1 |
| - 1. Informieren | 57 |
| - - 1. über SAPV Arbeit | 44 |
| - - 1. early integration | 9 |
| - 1. Zielerfassung, Auftrag, Behandlung | 70 |
| - 1. Bedarfserhebung | 129 |
| - - 1. Erfahrung/Fingerspitzengefühl | 68 |
| - - - 1. Sensible Arbeitsweise | 23 |
| - 1. Abschluss, Ausschleusung, Pausieren | 24 |
| - 1. Fallbesprechungen | 41 |
| 1. Gelungene Versorgung | 78 |
| - 1. Aufklären | 6 |
| - 1. Vermitteln | 36 |
| - 1. Schwelle | 31 |
| - 1. Aushalten | 25 |
| - 1. Sicherheitsempfinden | 88 |
| - 1. Individualität | 118 |
| - - 1. zum individuellen Fall informieren | 29 |
| - 1. "Beziehungsqualität" | 89 |
| - 1. Zeit haben | 61 |
| - 1. Fachkompetenz | 31 |
| - 1. Antizipation | 28 |
| - 1. Ganzheitlichkeit, umfassende Zuständigkeit, Komplexität | 53 |
| 1. Patienten: Bedürfnisse | 7 |
| - 1. Vertrauen | 38 |
| - 1. Würde | 51 |
| - 1. Autonomie, Mitbestimmung | 124 |
| - 1. Empowerment Patient | 10 |
| - 1. Normalität/ Alltag | 18 |
| - 1. Zu Hause bleiben | 112 |
| - 1. Lebensqualität | 36 |
| - - 1. Wünsche, Ziele | 36 |
| 1. Körperliche Aspekte | 0 |
| - 1. körperlich - Symptomkontrolle | 115 |
| 1. Psychische Aspekte | 0 |
| - 1. psychisch - Entlastung | 77 |
| 1. Soziale Aspekte | 0 |
| - 1. Dinge ordnen | 18 |
| - 1. sozial - Teilhabe | 31 |
| - 1. organisatorische Entlastung | 12 |
| - 1. Fürsorge für Zugehörige/ Bedürfnisse | 105 |
| - - 1. Zeit haben | 17 |
| - - 1. Entlastung (psychosozial, organisatorisch) | 64 |
| - - 1. Vorbereitung und Nachsorge/ Anschlussversorgung | 53 |
| - 1. Einbindung, Beteiligung in Versorgung | 45 |
| - - 1. Empowerment Zugehöriger | 29 |
| 1. Palliativsituation | 0 |
| - 1. Spiritualität | 106 |
| - 1. Seelsorge - extern | 42 |
| - 1. Ethisch | 20 |
| Sterben, Tod | 139 |
| 1. Zitate | 80 |

- 1. **Interviews with patients and relatives**

| **Codes** | **Number of Codings** | |
| --- | --- | --- |
| 1. zur Person Patient/Nahestehender | | 4 |
| - 1. Erkrankung des Patienten | | 107 |
| 1. Palliativstation | | 0 |
| - 1. Aufnahme | | 29 |
| - 1. Aufklären über Diagnose, Zustand | | 12 |
| - 1. Untersuchungen | | 3 |
| - 1. Therapieangebote | | 33 |
| - 1. Kompetenz | | 6 |
| - 1. Besonderheiten Palliativstation | | 19 |
| - - 1. Umgebung, Ruhe | | 25 |
| 1. SAPV | | 0 |
| - 1. Versorgungsverlauf | | 69 |
| - 1. Ziele der SAPV-Arbeit | | 5 |
| - 1. Kompetenz | | 29 |
| - 1. Zufriedenheit mit SAPV | | 22 |
| - 1. Leistungserbringer außer SAPV | | 73 |
| 1. Beziehungsqualität | | 26 |
| - 1. den Menschen sehen | | 56 |
| - 1. Vertrauen | | 24 |
| - 1. Normalität, Fröhlichkeit | | 16 |
| 1. Sicherheitsgefühl | | 28 |
| - 1. Verfügbarkeit | | 94 |
| - 1. Antizipation + Informieren | | 37 |
| - 1. Empowerment | | 34 |
| - 1. gegenseitiges Kennen | | 15 |
| 1. Patient/in, Erkrankte/r | | 0 |
| - 1. Lebensqualität | | 167 |
| - - 1. Kontakt zu Angehörigen | | 31 |
| - 1. Individualität | | 18 |
| - 1. Selbstbestimmung | | 86 |
| - 1. Normalität erhalten | | 20 |
| - 1. Zeit nehmen/Zeit haben | | 23 |
| - 1. Zu Hause sein | | 120 |
| - - 1. SAPV | | 14 |
| - - 1. Palliativstation | | 25 |
| - - 1. Entscheidung zum Krankenhausaufenthalt | | 28 |
| - - 1. stat. Hospiz | | 16 |
| - 1. Entlastung | | 14 |
| 1. Körperliche Aspekte | | 0 |
| - 1. körperliches Befinden, Symptome | | 196 |
| 1. Psychische Aspekte | | 0 |
| - 1. psychisches Befinden, Symptome | | 148 |
| 1. Soziale Aspekte | | 0 |
| - 1. Anschlussversorgung | | 14 |
| - 1. soziale Dimension | | 100 |
| - 1. umfassende Fürsorge | | 53 |
| - - 1. Hilfsmittel | | 52 |
| - - 1. Koordination, Kooperation | | 55 |
| - 1. Nahestehende/r, Angehörige/r | | 0 |
| - - 1. Belastung/Entlastung Nahestehende | | 134 |
| - - 1. Beteiligung in Versorgung | | 133 |
| 1. Palliativsituation | | 0 |
| - 1. spirituelle Aspekte | | 54 |
| - 1. Sterben, Palliativsituation | | 156 |
| - 1. Palliativsituation der Bezugsperson | | 98 |
| 1. Zitate | | 126 |
