# Supplementary material for: Key aspects of psychosocial needs in palliative care - a qualitative analysis within the setting of a palliative care unit in comparison with specialised palliative home care
Source: BMC Palliat Care. 2023 Jul 21;22:100. doi: 10.1186/s12904-023-01227-z (PMC10360287; doi:10.1186/s12904-023-01227-z)
Supplement: Supplementary file 2 — Supplementary Material 2 [file 12904_2023_1227_MOESM2_ESM.docx]

## COREQ (COnsolidated criteria for REporting Qualitative research) Checklist

**Article:**

**Key aspects of psychosocial needs in palliative care
-a qualitative analysis within the setting of a palliative care unit in comparison with specialized palliative home-care.**

**Authors:**

Cathrin Michel, Hannah Seipp, Katrin Kuss, Michaela Hach, Andrea Kussin, Jorge Riera-Knorrenschild and Stefan Boesner

**Journal:** BMC Palliative Care

| **Item No.** | **Guide question/description** | **Reported in Section (Page)** |
| --- | --- | --- |
| Domain 1: **Research team and reflexivity** | | |
| Personal Characteristics | | |
| 1. Interviewer/ facilitator | Which author/s conducted the interview or focus group? | Recruitment and data collection (p.7) |
| 1. Credentials | What were the researcher’s credentials? E.g. PhD, MD | Recruitment and data collection (p.7) Data analysis (p.8) |
| 1. Occupation | What was their occupation at the time of the study? | Declarations (p.33) |
| 1. Gender | Was the researcher male or female? | Recruitment and data collection (p.7)  Data analysis (p.8) |
| 1. Experience and training | What experience or training did the researcher have? | Recruitment and data collection (p.7)  Data analysis (p.8) |
| Relationship with participants | | |
| 1. Relationship established | Was a relationship established prior to study commencement? | Recruitment and data collection (p.7) |
| 1. Participant knowledge of the interviewer | What did the participants know about the researcher? e.g. personal goals, reasons for doing the research | Recruitment and data collection (p.7) |
| 1. Interviewer characteristics | What characteristics were reported about the interviewer/facilitator? e.g. Bias, assumptions, reasons and interests in the research topic | Declarations (pp.32/33) |
| Domain 2: **study design** | | |
| Theoretical framework | | |
| 1. Methodological orientation and Theory | What methodological orientation was stated to underpin the study? | Setting and Study design (p.6) |
| Participant selection | | |
| 1. Sampling | How were participants selected? e.g. purposive, convenience, consecutive, snowball | Recruitment and data collection (p.7) |
| 1. Method of approach | How were participants approached? e.g. face-to-face, telephone, mail, email | Recruitment and data collection (p.7) |
| 1. Sample size | How many participants were in the study? | Results, Table 1 (p.10) |
| 1. Non-participation | How many people refused to participate or dropped out? Reasons? | Results (p.10) |
| Setting | | |
| 1. Setting of data collection | Where was the data collected? e.g. home, clinic, workplace | Recruitment and data collection (p.7) |
| 1. Presence of non-participants | Was anyone else present besides the participants and researchers? | Recruitment and data collection (p.7) |
| 1. Description of sample | What are the important characteristics of the sample? e.g. demographic data, date | Results, Tables 1, 2, 3 (pp. 10/11) |
| Data collection | | |
| 1. Interview guide | Were questions, prompts, guides provided by the authors? Was it pilot tested? | Supplemental material 1 |
| 1. Repeat interviews | Were repeat interviews carried out? If yes, how many? | Results (p.10) |
| 1. Audio/visual recording | Did the research use audio or visual recording to collect the data? | Recruitment and data collection (p.7) |
| 1. Field notes | Were field notes made during and/or after the interview or focus group? | Recruitment and data collection (p. 7) |
| 1. Duration | What was the duration of the interviews or focus group? | Results (p.10) |
| 1. Data saturation | Was data saturation discussed? | Recruitment and data collection (p.7/8) |
| 1. Transcripts returned | Were transcripts returned to participants for comment and/or correction? | Recruitment and data collection (p.8) |
| Domain 3: **analysis and findings** | | |
| Data analysis | | |
| 1. Number of data coders | How many data coders coded the data? | Data analysis (p.8) |
| 1. Description of the coding tree | Did authors provide a description of the coding tree? | Supplemental material 2 |
| 1. Derivation of themes | Were themes identified in advance or derived from the data? | Data analysis (p.9) |
| 1. Software | What software, if applicable, was used to manage the data? | Data analysis (p.9) |
| 1. Participant checking Reporting | Did participants provide feedback on the findings? | Data analysis (p.9) |
| Reporting | | |
| 1. Quotations presented | Were participant quotations presented to illustrate the themes / findings? Was each  quotation identified? e.g. participant number | Results (pp.12-24) |
| 1. Data and findings consistent | Was there consistency between the data presented and the findings? | Results (pp.12-24) |
| 1. Clarity of major themes | Were major themes clearly presented in the findings? | Results (pp.12-24) |
| 1. Clarity of minor themes | Is there a description of diverse cases or discussion of minor themes? | Results (pp.12-24),  Discussion (pp.25-29) |
